# The Effect of Ethical Leadership on Nurse Bullying, Burnout, and Turnover Intentions

**DOI:** 10.1155/2024/3397854

**Published:** 2024-10-12

**Authors:** Jason R. Lambert, Lee W. Brown, Thanayi A. Lambert, Caleigh Torres Nava

**Affiliations:** ^1^College of Business, Texas Woman's University, Denton 76204, Texas, USA; ^2^College of Nursing and Health Innovation, University of Texas at Arlington, Arlington 76019, Texas, USA

**Keywords:** burnout, ethical leadership, turnover intentions, United States, workplace bullying

## Abstract

The bullying of nurses by patients, doctors, and employees is common in the healthcare industry. Nurses who are bullied are more likely to experience burnout, and nurses who experience burnout are more likely to intend to quit. However, few studies investigate how leadership can mitigate workplace incivility and nurse bullying as a way to improve nurse retention. A cross-sectional study was conducted using a sample of 216 nurses recruited from various regions across the United States from different specialties. A moderated mediation model using path analysis was used to examine the relationships between bullying, burnout, and ethical leadership in predicting intentions to stay. Bullying significantly and positively related to burnout (*β* = 0.22, *p*=0.02), and burnout significantly and negatively related to intent to stay (*β* = −0.18,*p*=0.01). Perceived ethical leadership predicted intentions to stay (*β* = 0.62, *p*=0.00), and ethical leadership moderated the effect of bullying on burnout (*β* = 0.20, *p*=0.03). The results of our study also suggest that nurses are less likely to quit when ethical leadership is present, and ethical leadership weakens the effect of bullying on burnout.

## 1. Introduction

Nurses are a key part of the healthcare system; they have the majority of direct interactions with patients, and it is estimated that by the next decade over 13 million nurses will be needed worldwide [1]. In the United States, there will be a national shortage of 63,720 full-time registered nurses for nursing roles by the year 2030 [2]. Additionally, the health systems of all 63 countries of the World Health Organization European Region have been adversely affected [3], and a shortage of more than 100,000 nurses is expected in Australia by the year 2025 [4]. This shortage is, in part, due to a variety of factors including a decline in nursing faculty, an aging population, an aging nursing workforce, burnout, staffing ratios, and verbal abuse in healthcare settings [4]. Many new nurses graduating and beginning their careers are not staying in the profession as a whole for long [5]; in addition, fewer nurses are becoming certified [6–8]. Additional factors contributing to this shortage include workplace environment, team dynamics, leadership style, organizational commitment, and turnover [7]. As this historical shortage persists in the United States [9, 10], patient care suffers, and the remaining nurses tend to be overworked, which leads to more negative experiences for those remaining.

In recent years, events such as the introduction of the Affordable Health Care Act in the U.S. have resulted in a substantial increase in potential patients testing the capacity of healthcare providers [11]. Coupled with global health pandemics such as SARS, Ebola, and Covid-19 over the past few decades, first responders in healthcare have encountered additional strain that leads to burnout [12] and lower job engagement worldwide [13, 14]. For example, during the time of the COVID-19 pandemic, nurses in Australia and New Zealand experienced more stress and greater workloads [15, 16]. More than half of the 351 nurses in a study conducted in Oman during the pandemic reported high levels of job burnout [13]. This resulting burnout among nurses is high [17, 18], and it is an important variable that affects employee turnover intentions [19]. Therefore, it is critically important to assess the level of burnout among nurses and identify factors that can reduce it.

Turnover can be costly for organizations monetarily [20], but turnover among nurses has even greater implications for healthcare organizations because it negatively impacts patient outcomes [21] and healthcare resources [22]. Nursing shortages lead to negative outcomes including an increase in mortality, staff violence, accidents, and injuries [8], rates of nosocomial infections, patient mortality, patient falls, pressure ulcers, prolonged average hospital stay lengths, and additional healthcare costs [23], further validating the need for solutions to ameliorate it.

Prior research has shown that some main psychological predictors of turnover include job satisfaction [24, 25], organizational commitment, and intent to stay [24, 26], value attainment, and mood [25]. Being bullied at work [27], burnout [19, 28], and leadership style [29] have also been shown to influence employees' turnover intentions. In the context of nursing, inadequate staffing is another important predictor of turnover because it leads to greater physical exhaustion by nurses managing heavy workloads [28].

Bullying is one way in which experienced nurses control new nurses to meet objectives, but verbal abuse can be traumatic for new nurses [30] and lead to outcomes that are psychologically, physically, and emotionally harmful [31]. In prior research, bullying is “considered as the most chronic issue in the health-care sector” [32]: 2, and being placed in stressful and unethical situations such as this can lead to job burnout [33]. Moreover, nurses who experience bullying at work may leave the nursing profession altogether [34–36]. Although bullying and burnout can negatively affect outcomes for nurses and their patients, scant research addresses strategies to reduce bullying incidents, especially related to nursing [37].

Some research results suggest that leadership styles of nursing managers can impact nurse behavior [29]. Unfavorable leadership styles, such as toxic leadership, are positively related to turnover intentions [29] while favorable leadership styles, such as transformational leadership, are negatively related to turnover intentions [38]. Despite the call from scholars to redirect attention to ethical leadership to improve nursing and patient outcomes [39], empirical investigations on how it can impact a culture of workplace incivility and burnout are limited. Because ethical leadership can model ethical performance [37], we propose that ethical leadership will ameliorate workplace incivility, and possess a buffering effect on burnout that stems from bullying behavior.

In this study, we examined perceived ethical leadership among nurses working for various types of healthcare provider institutions, and the consequences of bullying and burnout on intentions to stay. Our aim is to understand some understudied causes of turnover intentions in nursing. Our study makes a significant contribution to the literature in the following ways. First, given the turnover crisis in the nursing profession [4, 8], we fill a void in the turnover intentions literature by investigating how an ethical leadership style can mitigate the effect of bullying on burnout. Furthermore, because ethical leadership is understudied in the nursing literature [32, 37], and bullying is prevalent among new nurses [40], we examine its effect on intentions to stay among nurses who experience burnout as a result of experiencing or observing bullying in the workplace. In line with prior research, we contend that bullying and burnout have negative effects on intentions to stay, and further the literature by proposing that perceptions of ethical leadership influence this relationship.

**Figure 1:**
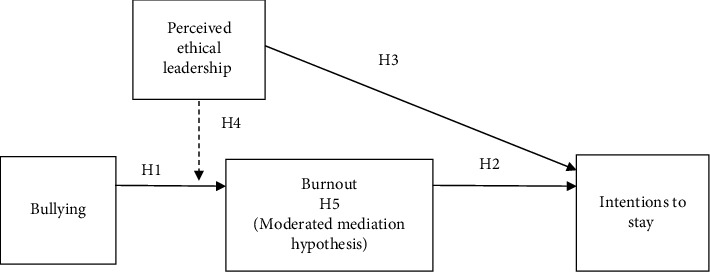
Conceptual model.

As shown in Figure 1, we hypothesize that bullying is directly related to job burnout of nurses. Further, we propose that job burnout is negatively related to intentions to stay. Also depicted by our model, we contend that leadership perceived to be ethical by nurses ameliorates the effect that bullying has on job burnout. Lastly, we propose the interaction effect between perceived ethical leadership and bullying on job burnout is indirectly related to intentions to stay resulting in a moderated mediation model.

### 1.1. Predictors of Turnover Intentions

Turnover intention refers to when an employee's motive is to leave a specific organization [41] and is a predictor of the actual act of turnover [42]. Job turnover among nurses has been an ongoing concern for many years [43] with the national average total hospital turnover rate being 19.5% as of 2022 [44]. In general, four types of variables have been studied extensively as predictors of turnover which include job training, job involvement, positive affectivity, and negative affectivity [45]. Positive and negative affectivity refer to dispositional states of pleasurable or unpleasurable engagement, respectively [46]. Justice, stress, and social support are variables associated with positive and negative affectivity used to research determinants of turnover [45], which include, but are not limited to, dimensions of supervisory support, role overload, role conflict, and perceptions of fairness [45]. Specific to the field of nursing, job turnover is exacerbated when nurses' have negative experiences in their work environment [47]. For example, other factors contributing to turnover include pandemic-related pressures [48], inadequate nurse staffing [49, 50], dealing with toxic leadership [29], and workplace violence [51]. In line with research on perceptions of fairness, bullying in the workplace is associated with high turnover intentions [52–54]. Viewed as a form of stress, burnout is also linked to intentions to quit and actual turnover [33]. As of recent, nurses specifically are leaving the nursing field due to emotional exhaustion brought on by high job demands, low job control, and role overload due to caring for COVID-19 patients [55], that also contributes to lower overall psychological well-being among nurses [48]. Besides dispositional effects on turnover intentions, perceptions regarding the nursing profession and the negative public image of their organization also affects nurses' job satisfaction which in turn influences their decision to quit their job or the profession entirely [51, 56].

### 1.2. Nursing Context

For our study, we focus on bullying and how it leads to both burnout and nurse intentions to stay because healthcare organizations are especially susceptible to bullying behaviors [30, 57]. There is evidence to suggest this may be because nurses are taught to bully others as an organizational cultural norm [58], and that healthcare systems traditionally operate using paternalistic styles of leadership that can lead to the oppression of nurses [58], exacerbated by authoritarian management practices [59]. Bullying is the act of aggressive behavior toward an individual repeatedly over time [60] which may also violate employees' civil rights [61]. Due to both the nature of their work environments and hospital group dynamics, nurses work under unique conditions that can be tied to turnover, including the experience of both physical and psychological effects. Physical conditions experienced by nurses from doctors, patients, and co-workers include “invasion of personal space, shoving and blocking the way, threat of violence, physical abuse, or actual abuse” [57]: 140. Patients in facilities where nurses are bullied can also receive poor nursing care or experience adverse events [62, 63]. We suggest that the nurse manager can play a key role in mitigating these bullying behaviors as a way to increase nurse retention and improve patient outcomes. Specifically, we study the effect that the ethical leadership of the nurse manager has on these scenarios.

### 1.3. Bullying and Job Burnout

Job burnout occurs when employees experience chronic stress resulting from their job [64] and is caused by various stressors which may include a number of physical or psychological issues nurses experience in their workplaces [65]. A key dimension of burnout is exhaustion [33], which is defined in the nursing literature as a common feeling of physical and emotional overload experienced by nurses stemming from interactions with co-workers and patients [66]. A work environment where bullying occurs is a stressful environment where employees feel intimidated, abused, or insulted, resulting in a stressful work experience [32]. Workplace bullying among nurses can lead to a number of psychological and mental health outcomes and work behaviors such as quiet quitting [67], depression, suicidal ideation, post-traumatic stress disorder, deterioration in the quality of their work life [68], job dissatisfaction [51], and burnout [69]. Physical health symptoms experienced by bullied nurses include headaches, eating disorders, onset of chronic diseases, and sleep disturbances [40].

Emotional exhaustion, a dimension of burnout [33], can be exacerbated by bullying, which could have a greater effect on intent to leave than any other factor [35]. Bullying can manifest itself in unfair treatment or any sort of verbal or physical harassment and may greatly influence whether or not nurses stay with an organization [61]. Examples of bullying include, but are not limited to, verbal attacks, intimidations, and withholding support [70] and its effect on employees shares similar characteristics to symptoms of burnout including anxiety and depression symptoms [33, 71, 72]. Further, prior research has shown that nurses experience burnout in climates where workplace bullying occurs [73]. In line with prior research, we contend that bullying is another prevalent factor that explains intentions to quit as it influences feelings of burnout.

**Hypothesis 1 .**

Bullying is positively related to burnout.

### 1.4. The Effect of Burnout on Intentions to Stay

Some of the global nursing shortage can be attributed to burnout, as increasingly more countries report how it has directly impacted turnover across their health systems [50]. Since nurses are likely to experience job burnout [74], the relationship between burnout and turnover must be further explored, especially since nurses have ongoing interaction with patients and their visitors, and frequent encounters with work stressors [75]. Burnout is considered a stress phenomenon, linked to lower job performance and poor health outcomes [33, 76], physical and mental health outcomes including psychological and physiological fatigue [75], impaired short-term memory and cognitive decline [77], alcohol and drug use [76], and disintegration of family and social relationships [74, 78, 79]. Under stressful circumstances associated with burnout, employees are less committed to the organization [80], become exhausted, and struggle to continue working. Some recent examples of stressful circumstances that relate to burnout and turnover among nurses include, but are not limited to, inadequate staffing [50], anxiety, and fear stemming from caring for patients during the COVID-19 pandemic [81]. Consequently, nurses experiencing burnout seek other jobs [82] and are more inclined to quit [75]. Not only is nurse burnout related to greater turnover [51, 83], burned-out nurses may no longer communicate effectively with others, engage in behaviors of workplace incivility [83], and experience physical and mental health challenges. Burned-out nurses will also express indifference toward patients [84] which can jeopardize their safety [21].

**Hypothesis 2 .**

Burnout is negatively related to intentions to stay.

### 1.5. The Effect of Ethical Leadership on Employees and Organizations

The type of leadership employees receive can greatly influence performance, especially in an environment like that of nursing. Ethical leadership is an observable expression from leaders of support for behavior deemed appropriate for followers that may be enacted through relationship building [85], communication, decision-making, and reinforcement [86]. Various studies have shown that ethical leadership creates an ethical environment conducive for reducing moral distress, thereby improving job satisfaction and absenteeism [87]. However, among the few studies on ethical leadership in the nursing literature that do exist, the number of quantitative studies is lacking [88].

Leaders in an organization set the tone for employees in terms of establishing a support system, the work environment, and acceptable behaviors. In fact, one of the most powerful methods to promote ethics in healthcare and the nursing practice is to role model ethical performance on the managerial level [37]. Leaders, through displaying and modeling ethical leadership, foment an ethical environment for nurses. Working in an ethical environment is especially important in a healthcare environment; especially at the nurse level, given that they interact the most frequently with patients.

### 1.6. Ethical Leadership and Turnover Intentions

In environments where nurses are “faced with obstacles that force them to act against their ethical beliefs, they feel discomfort, dissatisfaction, and frustration” [89]: 5, which can increase intentions to quit. An ethical environment allows nurses to feel supported and operate in an environment that is conducive to a higher quality of patient care. Moreover, ethical leadership influences the behavior of employees through its influence on the work climate [90]. However, many nurses report that they feel unsupported by their leaders [37] and some nurse leaders admit that ethical practice should be emphasized more as a part of organizational support [39]. However, the literature regarding the role of ethical leadership in nursing has recently been sparse [37]. For these reasons, we expect ethical leadership to be immensely important in the nursing field, which can prove beneficial for healthcare systems via increased job satisfaction for nurses and better patient outcomes [39]. Medical practices may receive outsized gains for attracting and developing ethical leaders throughout their organization due to the difficult work climate that nurses face on a day-to-day basis [91]. Furthermore, results from prior research suggest that a climate perceived as ethical helps prevent burnout [92] and strengthens organizational commitment [92–94], and job satisfaction [95–97], both predictors of turnover intentions [56]. For this reason, we expect to find that strong ethical leadership has a direct relationship with intention to stay.

**Hypothesis 3 .**

Perceived ethical leadership is positively related to intentions to stay.

### 1.7. The Interaction Between Perceived Ethical Leadership and Bullying

While we suggest that bullying [27] and burnout [98] will increase employees' intentions to leave an organization, we propose that the employment and development of ethical leaders in the organization can mitigate those intentions. Ethical leadership in place decreases employee anxiety about their job and increases employee behavior aligned with ethical principles [99]. Ethical leaders are both moral persons and moral managers [100]. Not only do they need to be moral individuals, but also they must apply that morality in the workplace. Brown and colleagues [86] define ethical leadership as “the demonstration of normatively appropriate behavior through personal actions and interpersonal relationships, and the promotion of such conduct to followers through two-way communication, reinforcement, and decision-making (p. 120).” Prior research has utilized social learning theory to explain how ethical leadership can mitigate negative workplace environments [101, 102]. This is welcome news for healthcare providers and nurses in particular because work environments in healthcare organizations have been shown to create a climate that leads to burnout [103].

Ethical leaders set a standard for what moral behavior is like in the workplace [104]. Employees with ethical leaders learn what is and what is not appropriate behavior in the organization through their leaders. In addition to the likelihood that bullying would decrease under ethical leaders due to an increased ethical climate [105], employees reporting to ethical leaders will be much less likely to assume bullying behavior is normal and appropriate workplace behavior. Evidence suggests that employees with ethical leaders will develop the ability and willingness to confront and address the conflict behavior [106] rather than assuming that exiting the organization is their only way out. For example, Islam and colleagues [32] found that the presence of ethical leadership positively influenced employee voice behavior and negatively affected bullying. In addition to the ethical role modeling and increased psychological safety that employees feel when working for ethical leaders, these ethical leaders have also been found to increase employees ability to deal with relationship conflict situations in the workplace by increasing employees' ability to create resolution [101]. In addition to social learning, the way that leaders design the work environment has been found to be a key mechanism for ethical leaders to decrease workplace bullying [107]. Prior research indicates that ethical leaders influence both the work climate that an employee works in and influences the behaviors and abilities of employees directly. For these reasons, we expect ethical leadership to moderate the effect that bullying has on employee burnout.

**Hypothesis 4 .**

Perceived ethical leadership moderates the effect of bullying on burnout such that nurses will experience less burnout from bullying when ethical leadership is high compared to when it is low.

Thus far we have proposed that bullying is positively related job burnout (i.e., Hypothesis 1 above), and that this relationship is moderated by perceived ethical leadership (i.e., Hypothesis 4 above). We also proposed that perceived ethical leadership is positively related to intentions to stay (i.e., Hypothesis 3 above). As prior research suggests that job burnout predicts turnover intentions, we contend that perceived ethical leadership signals to nurses messages of support regarding bullying prevention which, in turn, improve their feelings of job burnout, and ultimately intentions to stay. Thus not only do we propose that job burnout is related to intentions to stay (i.e., Hypothesis 2 above), we also propose that a conditional indirect effect exists for bullying and perceived ethical leadership on intentions to stay through job burnout. Based on prior research regarding the mediating role of burnout [79], this type of model is a moderated-mediation model [108, 109] resulting in our final hypothesis (full model shown in Figure 1).

**Hypothesis 5 .**

Bullying is related to intentions to stay via conditional indirect effects, such that its relationship with intentions to stay is moderated by perceived ethical leadership and mediated by job burnout.

## 2. Materials and Methods

### 2.1. Participants

A total of 216 nurses from various regions across the Unites States were recruited from different specialties to participate in the current research study in exchange for modest compensation. One hundred were identified through an email listserv managed by the Association of Women's Health, Obstetric, and Neonatal Nurses (AWHONN) and followed by nurses in obstetrics and women's health as their primary field. The remaining 116 were recruited using an online surveying agency (TurkPrime's Prime Panels platform: https://www.turkprime.com/LaunchedSurvey/PrimePanels) that commissions large-scale stratified samples. By recruiting participants from a wide array of online sources, this service allows researchers to set a priori demographic quotas for sampling. The panel was set to ensure the recruitment of participants from the nursing field.

### 2.2. Measures

All of the scales of measurement used for the current study were adapted from those published in peer-reviewed journals and have a reliability Cronbach's alpha coefficient greater than 0.80.

#### 2.2.1. Intentions to Stay

The dependent variable intentions to stay were measured using a four-item five-point Likert scale [110] used to measure intent to stay. For the current study *α* = 0.89. Sample items include “I would like to leave my present employer” and, “I plan to stay with my present employer as long as possible.”

#### 2.2.2. Bullying

The workplace incivility/bullying culture scale [111] was adapted to measure the independent variable bullying. It includes 12 items and uses a five-point Likert scale. The scale yielded a coefficient *α* = 0.96 for the current study. The survey begins with the following incomplete question to be completed by the 12 listed items to total 12 questions: “During the past year were you ever in a situation in which any of your supervisors or coworkers…” Sample items used to complete the question asked of participants include “Gave you hostile looks, stares, or sneers” and, “Made jokes at your expense.”

#### 2.2.3. Burnout

Malach-Pines' [112] short version of burnout measure was used to measure the mediating variable job burnout of participants in the current study. It includes 10 items using a seven-point Likert scale and yielded a coefficient *α* = 0.92. Participants are asked an introductory question “When you think about your work overall, how often do you feel the following?” Sample items that follow the introductory question for participants to answer include “Hopeless”, and “Difficulties sleeping.”

#### 2.2.4. Perceived Ethical Leadership

We measured perceptions of ethical leadership using the 10 item five-point Ethical Leadership Scale that Brown and colleagues [86] developed. For the current study *α* = 0.96. Sample items include, “Conducts his/her personal life in an ethical manner,” and “Sets an example of how to do things the right way in terms of ethics.”

#### 2.2.5. Controls

Age, race and organizational tenure were originally entered as control variables, and because they significantly did not have an effect on the primary variables of interest we removed them from the main statistical analysis.

### 2.3. Procedures

Online surveys were used to collect the data for this study. Participants replied to a link sent to their email address after having signed up to be a part of the research study either through their listserv announcement or as part of the Prime Panels platform. Upon clicking on the link, participants were redirected to an introductory web page where they provided consent before completing a battery of questions including surveys that measured the primary variables of interest in the current study dispersed among other surveys that measured various work attitudes, perceptions, beliefs, and basic demographic information.

### 2.4. Statistical Analysis

**Figure 2:**
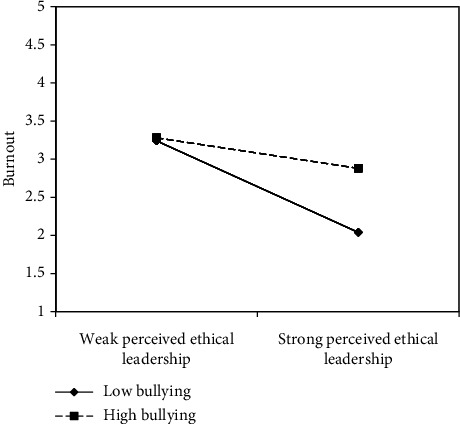
Graphed interaction effect between bullying and perceived ethical leadership on burnout.

A moderated mediation model using path analysis in Mplus v.8 was conducted to examine proposed relationships [113] as seen in Figure 2. A moderated mediation path model is a statistical technique used to examine how the relationship between two variables (independent and dependent variable) is influenced by a third variable (moderator) through a mediating variable [114, 115]. In this model, the mediator (burnout) lies between the independent and dependent variables and explains part of the effect of the independent variable on the dependent variable. The moderating variable (ethical leadership) influences the strength or direction of the relationship between the independent variable and the mediator, ultimately affecting the indirect effect on the dependent variable. The current model examined the interaction between bullying, burnout, and ethical leadership in predicting intentions to stay. The primary purpose of the model was to examine how job burnout mediated the relationship between bullying and intentions to stay. In addition, the interaction effect between bullying and perceived ethical leadership on job burnout was tested to examine the mediation effect further.

## 3. Results and Discussion

### 3.1. Results

Participants with missing data related to the variables being investigated were removed resulting in 184 valid responses (*N* = 184); a response rate of 85.2%. Respondents were 92% female with an average age of 40.2 years (SD = 11.95), and most were White (84%), followed by Black (7.4%), Hispanic/Latinx (6.8%), and American Indian (1.9%).

Table 1 shows the means, standard deviations and bivariate correlations for all study variables. It should be noted that the path model was just-identified, thus, model fit indices were not useful (i.e., RMSEA = 0.00, CFI and TLI = 1.00). Our first and second hypotheses were supported as expected indicating that bullying significantly and positively relates to burnout (*β* = 0.22, *p*=0.02), and burnout significantly and negatively relates to intent to stay (i.e., intentions to quit) (*β* = −0.18, *p*=0.01), respectively. Hypothesis 3 which proposed that perceived ethical leadership predicts intentions to stay was also supported with significance (*β* = 0.62, *p*=0.00), and our fourth hypothesis proposing that ethical leadership moderates the effect of bullying on burnout was supported (*β* = 0.20, *p*=0.03). The graph of the interaction in Figure 2 indicates the direction hypothesized with perceived ethical leadership mollifying the effect of bullying on burnout. Our fifth and final hypothesis proposed a moderated mediation model. Although there were significant paths from bullying to burnout and from burnout to intent to stay, and the interaction term was significant, the indirect effect of bullying on intentions to stay was not significant; hence Hypothesis 5 was not supported (*β* = −0.04, *p*=0.08).  Table 2 presents the results and coefficients for the observed variables.

### 3.2. Discussion

There is an ongoing shortage of skilled nurses across many high income countries. The key components of nursing work of providing care are challenging and emotionally trying, which have an impact on the shortage and the ability of the industry to attract and retain nurses [116], and the resulting quality of patient care [28]. Prior research has established that bullying relates to burnout [23, 69, 116–118], yet there is limited research that examines solutions to mitigate this relationship, and how nurse leaders, specifically, can address this cultural phenomenon. There is limited research that investigates the effect of ethical leadership on nursing outcomes [37], and scant research in the nursing literature addresses how various styles of leadership can mitigate the effect of bullying on burnout and turnover intentions, specifically. The results of the current study contribute to the literature and to solutions for mitigating burnout and turnover among nurses in a number of ways.

The results of the current study align with prior research suggesting that bullying relates to burnout, and that burnout relates to turnover intentions [23, 118]. The results also suggest that bullying has a main effect on turnover intentions. However, we found no indirect effect for bullying on turnover intentions through burnout in the present study, compared to prior studies with nurses working in Taiwan [23] or Korea [118]. This could be due to cultural differences and how workplace culture is perceived differently by nurses in the United States compared to nurses from other countries. For example, based on Hofstede's dimension of national cultures [119], the United States ranks lower than Taiwan and Korea on the dimensions of power distance and higher on the dimension of individualism. Power distance is defined as the degree to which employees prefer a consultative or participative style of leadership (i.e., low ranking) versus an autocratic style of leadership (i.e., high ranking) [119]. Individualism refers to the degree in which a person views their connection to their society on a spectrum of either having loose ties to others and looking after one's self (i.e., high ranking), or valuing strong bonds with their community and having a great sense of loyalty to their society (i.e., low ranking) [119]. Employees in the U.S. who prefer a consultative style of leadership (i.e., low power distance) may be less apprehensive about expressing their opinions, attitudes, and beliefs with leadership compared to employees in other countries where a more autocratic style of leadership is preferred (i.e., high power distance). Also, employees in the U.S. who are more likely to hold individualistic ideals (i.e., high individualism) may be more likely to challenge the “status quo” compared to employees from countries where collectivism ideals (i.e., low individualism) are the norm. We posit that this perspective may weaken or eliminate the indirect effect that bullying has on turnover intentions. This is because their perceived ability to express concern about bullying may serve as a coping mechanism for reducing burnout, thereby weakening the relationship. More research about the relationship between bullying, burnout, and turnover intentions among nurses in the U.S., and its comparison across multiple countries may offer a more comprehensive understanding of this phenomenon.

Suggestions for mitigating the effects of bullying and burnout on turnover have been proposed in prior research [23, 118], but there is limited empirical evidence that these solutions work. Although prior research has investigated the relationship between leadership, bullying, burnout, and turnover intentions, not all leadership styles have been empirically examined as a means to mitigate the negative effects of this relationship. Laschinger and colleagues [120] conducted a study with results to suggest that authentic leadership negatively affects workplace bullying. Another study, qualitative in nature, suggested that transformational leadership attributes are important for establishing a positive hospital work environment [121]. We responded to the call for a greater examination of ethical leadership as an effective tool to manage nursing outcomes in healthcare [37, 118], and specifically the call to propose interventions to mitigate the effect of bullying on burnout [69]. Not only did the current study results suggest that ethical leadership has a positive main effect on intentions to stay, the results also show that ethical leadership ameliorates the effect of bullying on burnout. Providing ethical leadership training and employing nurses who demonstrate ethical leadership may be a useful strategy for mitigating the effect of bullying on burnout, two important predictors of turnover.

## 4. Limitations and Future Research

It is important to note a few limitations of this study. The current study used self-report measures where social desirability may have affected the results of the data in addition to mono-method bias as a result of common method variance [122]. Cross-sectional designs such as the kind used for our study also poses limitations on causal inferences regarding the relationships between the observed variables. Online data collection also can pose limitations for controlling environmental factors. However, prior research has established there is equivalence between the results of online survey methods and results produced using paper-and-pencil formats [123].

Although our study yielded significant findings, qualitative studies may offer additional explanations to why ethical leadership mitigates the effect of bullying on burnout and increases intentions to stay in the nursing field. The current study used the survey method to measure participants' perception about the ethical leadership of their nurse manager, which can limit the richness of the data collected. Research questions that address how followers identify ethical leadership in healthcare settings are warranted. For example, does it matter if perceived ethical leadership communication is verbal instead of written? Are there any effects for gender based on the identity of leaders or nurse managers? Building on prior research [124], should we investigate whether any effects exist for race based on the identity of leaders or nurse managers perceived as ethical? Future researchers should address these questions to explain the effect of different circumstances under which ethical leadership influences the behavior and attitudes of nurses.

## 5. Conclusions

The aim of the current study was to investigate the relationships between perceived bullying, burnout, perceived ethical leadership, and turnover intentions. In line with prior research, the results suggest that nurses who get bullied are more likely to experience burnout, and nurses who experience burnout are more likely to intend to quit. Furthering this line of research, the results of our study also suggest that ethical leadership weakens the effect of bullying on burnout, and nurses are less likely to quit when ethical leadership is present [125–130].

### 5.1. Implications for Nursing Management Research

Our path analysis findings align with and extend previous research on workplace mental health outcomes in nursing. The significant relationship we found between bullying and burnout (*β* = 0.22, *p*=0.02) supports prior studies showing that workplace incivility and bullying contribute to emotional exhaustion and burnout among nurses [73, 116]. The moderating effect of ethical leadership on this relationship (*β* = 0.20, *p*=0.03) builds on work by Laschinger et al. [130] on authentic leadership, suggesting that multiple positive leadership styles may help buffer against the negative effects of bullying.

Furthermore, our results highlighting the importance of ethical leadership in predicting intentions to stay (*β* = 0.62, *p*=0.00) complement research on the nursing work environment. Studies have shown that supportive practice environments and ethical climates are associated with lower turnover intentions among nurses [96, 131]. Our findings suggest that ethical leadership may be a key factor in creating such positive work environments.

### 5.2. Implications for Nursing Management Practice

Developing a better understanding of ethical leadership's role in nursing has major implications for strategies to increase retention. There are instances when organizations go through periods of rough transitions when trying to improve their climate or culture. As leaders remove bully employees from their organization, ensuring there is ethical leadership in place may mitigate the degree of burnout experienced by nurses who are bullied. The first implication of our study is for healthcare administrators to recognize the importance of training nurse managers about ethical leadership principles and strategies. The study results suggest that this will help improve nurses' intentions to stay, and thereby reduce turnover in the field of nursing. Second, nurse managers who are perceived as ethical leaders have the effect of weakening the impact of workplace incivility on burnout. This should translate into better work outcomes for nurses and, in turn, improve health outcomes for patients. In line with prior recommendations for improving the nursing work environment [132], our results suggest that healthcare organizations should prioritize developing ethical leadership skills among nurse managers. This aligns with calls for creating positive practice environments to enhance nurse retention [133].

## Figures and Tables

**Table 1:** Descriptive statistics and bivariate correlations.

**Variable**		**Mean**	**SD**	**1**	**2**	**3**
1	Bullying	1.58	0.79			
2	Burnout	3.10	1.17	0.30^∗∗^		
3	Ethical leadership	0.13	1.10	−0.39^∗∗^	−0.42^∗∗^	
4	Intentions to stay	3.60	1.10	−0.36^∗∗^	−0.46^∗∗^	−0.61^∗∗^

*Note: N* = 184.

Abbreviation: SD, standard deviation.

^∗∗^ *p* < 0.01.

**Table 2:** Summary of path analysis predicting intentions to stay.

**Path**	**R** ^2^	***β***	**SE**
Burnout on	0.23^∗^		
Perceived ethical leadership		0.39^∗∗∗^	0.09
Bullying		0.22^∗^	0.09
Perceived ethical leadership X			
Bullying		0.20^∗^	0.09
Intentions to stay on	0.52^∗∗∗^		
Perceived ethical leadership		0.62^∗∗∗^	0.06
Burnout		−0.18^∗^	0.07

*Note: N* = 184.

Abbreviation: SE, standard error.

^∗^ *p* < 0.05; ^∗∗^*p* < 0.01; ^∗∗∗^*p* < 0.001.

## Data Availability

The data that support the findings of this study are available from the corresponding author upon reasonable request.
